# DNA Methyltransferase Inhibition Prevents Platinum-Induced Ovarian Cancer Stem Cell Enrichment

**DOI:** 10.1158/2767-9764.CRC-26-0149

**Published:** 2026-07-20

**Authors:** Truc T. Vuong, Riddhi Sood, Shu Zhang, Tara X. Metcalfe, Rena Y. Han, Kenneth P. Nephew, Heather M. O’Hagan

**Affiliations:** 1 Cell, Molecular and Cancer Biology Graduate Program, Indiana University School of Medicine, Bloomington, Indiana.; 2 Medical Sciences Program, Indiana University School of Medicine, Bloomington, Indiana.; 3 Indiana University Melvin and Bren Simon Comprehensive Cancer Center, Indianapolis, Indiana.; 4 Genome, Cell and Developmental Biology, Department of Biology, Indiana University Bloomington, Bloomington, Indiana.; 5 Department of Anatomy, Cell Biology and Physiology, Indiana University School of Medicine, Indianapolis, Indiana.; 6 Department of Obstetrics and Gynecology, Indiana University School of Medicine, Indianapolis, Indiana.; 7 Department of Medical and Molecular Genetics, Indiana University School of Medicine, Indianapolis, Indiana.

## Abstract

Platinum-based therapy is the standard first-line treatment for high-grade serous ovarian cancer (HGSC). However, most patients develop resistance and recurrence despite an initial response to therapy. Ovarian cancer stem cells (OCSC) are enriched in recurrent tumors and contribute to platinum resistance. These tumors also show promoter DNA hypermethylation, and DNA methyltransferase inhibitors (DNMTi) have been shown to restore sensitivity in platinum-resistant ovarian cancer cells. In this study, we demonstrated that combining DNMTi treamtent with platinum prevented the platinum-induced enrichment of OCSCs and identified nuclear factor kappa-light-chain-enhancer of activated B cells (NF-κB) and signal transducer and activator of transcription 3 (STAT3) signaling pathways as potential regulators of platinum-induced OCSC enrichment. STAT3 was active at baseline in ovarian cancer cells, and platinum treatment alone activated NF-κB while maintaining STAT3 activity. Platinum combined with DNMTi treatment decreased STAT3 activation, while still inducing NF-κB activation. Knockdown experiments demonstrated that the presence of both NF-κB and STAT3 was necessary for platinum-induced OCSC enrichment. Analysis of STAT3 and NF-κB subunit p65 CUT&RUN data showed increased binding in introns and intergenic regions in response to platinum. Additionally, DNMTi treatment enriched NF-κB binding at endogenous retroviruses, which correlated with changes in expression of nearby genes when DNMTi treatment was combined with platinum. We conclude that combining DNMTis with platinum modulates STAT3 and NF-κB activation and genomic binding, potentially influencing target gene expression and preventing platinum-induced enrichment of OCSCs.

**Significance:**

OCSCs promote relapse and chemoresistance in HGSC. We show that combining standard platinum-based therapy with DNMTi treatment prevents platinum-induced enrichment of OCSCs. Our findings support the use of DNMTis in combination with platinum as a strategy to reduce OCSC enrichment and platinum resistance in ovarian cancer.

## Introduction

Ovarian cancer is a deadly gynecologic malignancy and one of the leading causes of cancer-related deaths among women in the United States (1, 2). High-grade serous ovarian cancer (HGSC) is the most common subtype, accounting for 70% to 80% of all ovarian cancer cases (2). Due to the absence of effective early detection methods, most patients with HGSC are diagnosed at an advanced stage, when the disease has already metastasized, resulting in a 5-year survival rate of less than 30% (2, 3). The standard treatment involves debulking surgery to remove most of the tumor, followed by platinum- and taxane-based chemotherapy (2–4). Despite a high initial response rate, more than 80% of patients with ovarian cancer experience relapse, and recurrent tumors typically become resistant to platinum and other chemotherapies (4), posing a major obstacle to improving patient outcomes.

In various solid cancers, including ovarian cancer, cancer stem cells (CSC) survive chemotherapy, later driving tumor relapse and resistance, and are enriched in recurrent tumors (4–7). Aldehyde dehydrogenase (ALDH) is a well-established marker for ovarian cancer stem cells (OCSC), with ALDH-positive (ALDH+) OCSCs exhibiting stem-like features, including high tumor-initiating ability, spheroid formation, self-renewal, differentiation, and elevated expression of stemness genes such as *BMI1*, *OCT4*, and *NANOG* (8–12). Moreover, elevated levels of the ALDH isoform *ALDH1A1* are associated with poorer prognosis and platinum resistance in ovarian cancer (10). Studies have shown that ALDH+ OCSCs increase following platinum treatment in both *in vitro* and *in vivo* settings (9, 13), indicating that platinum-induced ALDH+ OCSCs contribute to relapse and resistance to platinum therapy.

In addition to the enrichment of OCSCs, increased DNA hypermethylation at gene promoters in recurrent ovarian cancer tumors has been reported, leading to the silencing of tumor-suppressor, proapoptotic, and developmental genes (5, 14–17). Global DNA methylation analysis in ovarian cancer cells revealed heightened abnormal promoter hypermethylation as the cells become resistant to platinum (16). DNA methyltransferase inhibitors (DNMTi) reduce this aberrant DNA hypermethylation and resensitize platinum-resistant ovarian cancer cells (5, 16). DNMTi treatment also induces the interferon (IFN) response in ovarian cancer by demethylating and upregulating endogenous retroviruses (ERV), thereby activating the cytosolic RNA-sensing pathway and eliciting an antitumor immune response (18, 19). Furthermore, we recently reported that combining DNMTi treatment with a PARP inhibitor induces viral mimicry and inflammatory responses with tumor-suppressor properties in ovarian cancer (19). Currently, approved clinical use of DNMTis remains limited to hematologic malignancies (20). Clinical trials of DNMTis in solid tumors, including ovarian cancer, have been conducted but have had limited success (20–23). Most trials involving DNMTis in ovarian cancer focused on tumors that were already resistant to platinum (21, 22); however, the effects of DNMTis when given before resistance onset are not well understood.

In this study, we investigate the effect of combining DNMTi and platinum treatments on the OCSC population. We demonstrate that whereas treatment with platinum alone increases ALDH+ OCSC cells, DNMTi treatment prevents the platinum-induced increase in ALDH+ OCSC cells. RNA sequencing (RNA-seq) analysis identifies signal transducer and activator of transcription 3 (STAT3) and nuclear factor kappa-light-chain-enhancer of activated B cells (NF-κB) signaling as potential pathways for further exploration of how DNMTi treatment blocks platinum-induced OCSC enrichment. STAT3 and NF-κB are transcription factors linked to stemness in various cancer types, including ovarian cancer (24–27). In this study, we demonstrate that the presence of both STAT3 and NF-κB is necessary for platinum-induced ALDH+ OCSC enrichment. Platinum treatment alone increases NF-κB activation and maintains baseline STAT3 activation. Conversely, combining platinum with DNMTi treatment decreases STAT3 activation while still inducing NF-κB activation. Interestingly, DNMTi treatment promotes NF-κB binding to ERVs, which correlates with changes in expression of nearby genes in samples treated with DNMTi and platinum. Overall, combining DNMTi treatment with platinum prevents platinum-induced enrichment of OCSCs by decreasing STAT3 activation and altering the NF-κB cistrome, likely affecting the expression of genes that regulate stemness. Importantly, these findings for the first time shed light on how DNMTi treatment blocks platinum-induced OCSC enrichment and provides a new rationale for using DNMTis as a potential first-line therapy with platinum to decrease OCSC enrichment and prevent recurrence and resistance in ovarian cancer.

## Materials and Methods

### Cell lines, culture, and treatments

HSGC cell lines OVCAR3 (RRID: CVCL_0465), PEO1 (RRID: CVCL_2686), OVSAHO (RRID: CVCL_3114), and OVCAR5 (RRID: CVCL_1628) were maintained at 37°C and 5% CO_2_ as described previously (13, 28). All cell lines were purchased from the ATCC. OVCAR3, OVCAR5, and OVSAHO cell lines were authenticated by the ATCC in 2018. The PEO1 cell line was authenticated by the ATCC in 2012. OVCAR5 and OVSAHO cells were last tested for *Mycoplasma* in 2017 using the Universal Mycoplasma Detection Kit (ATCC, 30-1012K). OVCAR3 and PEO1 were last tested for *Mycoplasma* in 2026. OVCAR3 and OVCAR5 cells were cultured in 1× DMEM (Corning, 10-013-CV), OVSAHO cells in RPMI (Corning, 10-040-CV), and PEO1 cells in RPMI supplemented with 2 μmol/L sodium pyruvate. All media were supplemented with 10% FBS (Gibco, A5670701) without antibiotics. All cell lines used in experiments were passaged fewer than 15 times. Filter-sterilized 1.67 mmol/L stock solutions of cisplatin (MilliporeSigma, 232120) were prepared in 154 mmol/L NaCl and stored at 4°C. Cells were treated with their respective IC_50_ doses of cisplatin (OVCAR3, 15 μmol/L; PEO1, 13 μmol/L; OVCAR5, 12 μmol/L; and OVSAHO, 4 μmol/L; refs. 28, 29). Stock solutions of decitabine (DAC; MilliporeSigma, A3656, 2 μg/μL) were prepared in water and stored at −80°C. Cells were treated with DAC at a final concentration of 100 nmol/L for 48 hours for OVCAR5 and OVSAHO or 72 hours for OVCAR3 and PEO1. The media containing fresh DAC were changed every 24 hours. For cisplatin and DAC dual treatment, cisplatin was added during the last 16 hours of DAC treatment. Treatment doses used for various assays are detailed in the figure legends.

### ALDEFLUOR assay and flow cytometry

Cells were cultured in 100-mm dishes (OVCAR3, 2 × 10^5^; PEO1, 3 × 10^5^; OVCAR5, 2 × 10^5^; and OVSAHO, 2.5 × 10^5^). After treatment, the ALDEFLUOR assay (StemCell Technologies, 01700) was performed according to the manufacturer’s instructions and as previously described (13). Briefly, 1 × 10^6^ cells were resuspended in ALDEFLUOR assay buffer (1 mL) containing BOPIDY-aminoacetaldehyde (BAAA) substrate, divided into two 500 μL aliquots, and the ALDH inhibitor diethylaminobenzaldehyde (DEAB; 5 μL) was added to one of the aliquots. Cells were incubated for 30 to 45 minutes at 37°C in the dark. After incubation, cells were centrifuged, resuspended in ALDEFLUOR assay buffer, and filtered through a 30-μm filter. Flow cytometry analysis was performed using LSRII flow cytometer (BD Biosciences, RRID: SCR_024398) and CytoFLEX LX (Beckman Coulter, RRID: SCR_025067) at Indiana University Flow Cytometry Core Facility. ALDH activity was measured using a 488-nm excitation laser, with the signal detected by the 530/30 (LSRII) and 525/40 (CytoFLEX LX) filters. For each experiment, 1 × 10^4^ events were analyzed. The ALDH+ percentage gate was set based on the DEAB-negative control ALDH+ gate, which contained 1% or fewer ALDH+ cells. Data analysis was conducted using FlowJo software (Becton, Dickinson & Company, RRID: SCR_008520).

### Spheroid formation assay

OVCAR3 (1 × 10^5^ in 60-mm dishes), OVCAR5 (5 × 10^5^ in 100-mm dishes), and OVSAHO (7.5 × 10^5^ in 100-mm dishes) cells were treated with cisplatin for 3 hours (OVCAR3, 7.5 μmol/L; OVCAR5, 6 μmol/L; and OVSAHO, 2 μmol/L), with or without 100 nmol/L DAC (OVCAR3, 72 hours; OVCAR5 and OVSAHO, 48 hours). After treatment, cells (OVCAR3, 2 × 10^3^; OVCAR5 and OVSAHO 1.5 × 10^4^) were transferred to a 24-well low-attachment plate (Corning, 3473) containing stem cell media, as we have described previously (13). Fresh stem cell media were added to each well every 3 days. Spheroids from OVCAR3 were imaged on day 7 of incubation using a Zeiss Axiovert 40 inverted microscope. The area of individual spheroids per well was measured using Fiji (version 2.16.0, RRID: SCR_002285). For each biological replicate, a threshold area of 10,000 μm^2^ was used. Single-cell aggregates were manually removed, and the remaining areas were considered spheroids. Spheroids from OVCAR5 and OVSAHO were imaged on day 14 of incubation using an EVOS FL Auto microscope (Life Technologies, RRID: SCR_026039). Then, reagent from the Cell Viability Assay Kit (Abcam, ab176748), which measures cell viability through intracellular esterase activity, was added directly to each spheroid well at a volume equal to the volume of media in the well. After 1 hour of incubation, the reagent-plus-media solution was transferred to an opaque 96-well plate. Cell viability (Ex/Em: 405/460 nm) was measured using a SynergyH1 plate reader (BioTek, RRID: SCR_019748). The graphs were plotted using data from three biological replicates for OVCAR3 and four replicates for OVCAR5 and OVSAHO.

### RNA-seq and data analysis

Total RNA was isolated from OVCAR3 and PEO1 cell pellets using the RNeasy Mini Kit (QIAgen, 74106) according to the manufacturer’s protocol. For OVCAR3 samples, libraries were prepared for sequencing using the Illumina TruSeq Stranded mRNA HT Library (Illumina, 20020594) as per the manufacturer’s instructions, followed by sequencing on the Illumina NextSeq 500 (RRID: SCR_014983). For PEO1 samples, 3 μg of RNA per sample was sent to Plasmidsaurus, where libraries were prepared and sequenced on the Illumina NovaSeq X Plus (RRID: SCR_024568) utilizing their proprietary RNA-seq pipeline. Sequencing read quality control for all samples was assessed with FastQC (version 0.11.9, https://www.bioinformatics.babraham.ac.uk/projects/fastqc/, RRID: SCR_014583). Read alignment was performed against the hg38 reference genome, followed by calculation of raw read counts using STAR (version 2.7.3a, RRID: SCR_004463; ref. 30). DESeq2 (version 1.36.0, RRID: SCR_015687) was used for differential expression analysis (31). Gene set enrichment analysis (GSEA) was performed using the Molecular Signature Database (MSigDB, RRID: SCR_016863) Hallmark gene sets and manually curated STAT3 and NF-κB target gene sets with the GSEA software (version 4.3.3, RRID: SCR_003199; refs. 32–35). STAT3 and NF-κB target gene sets were curated from MSigDB C3 transcription factor subcollection (36, 37), ENCODE (RRID: SCR_006793; refs. 38, 39), TRRUST (RRID: SCR_022554; ref. 40), ChEA (RRID: SCR_005403; ref. 41), ESCAPE (42), Jaspar (RRID: SCR_003030; refs. 43, 44), and MotifMap (45) databases (Supplementary Table S1). Normalized counts per million in bigWigs were generated using deepTools (version 3.5.6, RRID: SCR_016366) bamCoverage (46). Normalized read counts were calculated using DESeq2 plotCounts (31).

### Whole-cell, nuclear, and chromatin extraction and Western blot analysis

OVCAR3 (7 × 10^5^) and PEO1 (1 × 10^6^) cells were cultured in 150-mm dishes. After treatment, cell pellets were collected, with 30% of the volume set aside for whole-cell lysate preparation using QIAshredder (Qiagen, 79656) and 4% SDS. The remaining cell pellets were used for nuclear isolation with CEBN buffer [10 mmol/L HEPES, pH 7.8; 10 mmol/L KCl; 1.5 mmol/L MgCl_2_; 0.34 mol/L sucrose; 10% glycerol; 0.2% NP-40; 1× protease inhibitor (Thermo Fisher Scientific, A32965); and 1× phosphatase inhibitor (MilliporeSigma, P5726)]. The nuclear pellets were washed with CEB buffer (CEBN without NP-40) containing all inhibitors. Soluble nuclear extract was obtained by incubating the nuclear pellets in a soluble nuclear buffer (2 mmol/L EDTA, 2 mmol/L EGTA) with all inhibitors for 30 minutes on a rotator at 4°C. The remaining cell pellet represents the total chromatin fraction, which was lysed using QIAshredder and 4% SDS. Lysates were analyzed by Western blot. Relative densitometry of Western blots was quantified using Fiji and normalized to the density of GAPDH (for whole-cell lysates) and H3 (for whole-cell lysates and nuclear and chromatin extracts) loading controls. The results are shown as mean ± SEM.

### Secretome analysis

OVCAR3 cells (0.25 × 10^5^ per well) were cultured in a six-well plate. After treatment, 1 mL of media per condition was collected and centrifuged at 3,000 × *g* for 2 minutes at 4°C to remove debris. Then, 150 μL of supernatant was prepared for cytokine and chemokine analysis using the Human Cytokine/Chemokine 96-Plex Discovery Assay Array (HD96; Eve Technologies).

### DAC-treated media exchange

To prepare DAC-treated media, OVCAR3 cells (7 × 10^5^) cultured in 150-mm dishes were either mock-treated or treated with 100 nmol/L DAC for 72 hours. After 72 hours, the media were collected and centrifuged (3,000 × *g* for 2 minutes at 4°C) to remove debris. Naïve OVCAR3 cells were incubated in the collected media for 48 hours, with 15 μmol/L cisplatin added during the last 16 hours. The cells were then subjected to ALDEFLUOR assay and analysis as described above.

### Reverse transfection siRNA-mediated knockdowns

Knockdown (KD) of STAT3 and RELA was performed using siRNA as previously described (47). Briefly, 25 pmol of scrambled RNA control (scrRNA; Dharmacon, D-001810-10-05), a pool of four siRNAs targeting STAT3 (Dharmacon, L-003544-00-0005), or RELA (Dharmacon, L-003533-00-0005) were combined with OptiMEM media (Gibco, 31985-070) and Lipofectamine RNAiMAX (Thermo Fisher Scientific, 13778150) and added to 100-mm dishes. The mixture was incubated (room temperature, 20 minutes), and then OVCAR3 (7 × 10^5^) and PEO1 (7 × 10^5^) cells were seeded into wells containing the siRNA. After 24 hours, the media were removed, dishes were washed with PBS, and fresh media were added. Cells were treated for 16 hours with the IC_50_ value of cisplatin (OVCAR3, 15 μmol/L; PEO1, 13 μmol/L) and then collected for assays.

### RNA isolation and RT-qPCR

Total RNA was isolated from cell pellets using the RNeasy Mini Kit (Qiagen, 74106) according to the manufacturer’s protocol. For gene expression, cDNA was synthesized using the Maxima First Strand cDNA Synthesis Kit (Thermo Fisher Scientific, K1642), followed by amplification with gene-specific primers using the FastStart Essential DNA Probe Master (Roche, 06402682001) and FastStart Essential DNA Green Master (Roche, 06924204001). Relative gene expression was calculated using the Pfaffl method, with *PPIA* or *RHOA* as the housekeeping genes. Primer sequences are listed in Supplementary Table S2.

### Cleavage under targets and release using nuclease

Cleavage under targets and release using nuclease (CUT&RUN) was performed according to the CUTANA CUT&RUN protocol and as we previously described (48, 49). Briefly, after treatment, 1 × 10^6^ live OVCAR3 cells were washed, tethered to CUTANA concanavalin A magnetic beads (EpiCypher, 21-1401), and permeabilized in antibody buffer. Cells were then incubated overnight at 4°C on a nutator with antibodies against STAT3, p65, or mouse IgG and rabbit IgG, respectively. The slurry was washed with digitonin buffer and then incubated with pAG-MNase (EpiCypher, 15-1016) and CaCl_2_ for 2 hours on a nutator at 4°C. The slurry was then incubated in the stop buffer at 37°C for 10 minutes to halt MNase activity and release DNA fragments. Fragments from the supernatant were purified using the CUTANA DNA Purification Kit (EpiCypher, 14-0052) according to the manufacturer’s instructions. DNA concentration was measured using a Qubit fluorometer (Thermo Fisher Scientific, Q32866) and Qubit dsDNA Quantification Assay Kits (Thermo Fisher Scientific, Q32851). All DNA obtained from each sample was used to prepare an Illumina library using the NEBNext Ultra DNA Library Kit (New England Biolabs, E76450), following the manufacturer’s instructions, and sequenced on the Illumina NextSeq 2000 (RRID: SCR_023614). Two biological replicates were collected for each treatment per transcription factor.

### CUT&RUN sequencing analysis

Sequencing read quality control for all samples was evaluated using FastQC (version 0.12.1). Read alignment was performed against the hg38 reference genome with Bowtie2 (version 2.5.1, RRID: SCR_016368; ref. 50). Peaks were identified using MACS (version 3.0.3, RRID: SCR_013291; ref. 51), with IgG CUT&RUN samples serving as controls. The irreproducible discovery rate (IDR, RRID: SCR_017237) method (52) was applied to STAT3 and p65 CUT&RUN data to identify consistent peaks across the two biological replicates as per the ENCODE ChIP-seq analysis pipeline (Research Square rs.3.rs-3111932/v1; ref. 53), using IDR thresholds of 0.05 and 0.01, respectively. Blacklisted regions identified by Nordin and colleagues (54) were filtered out using BEDTools (version 2.31.0, RRID: SCR_006646; ref. 55). Motif analysis was performed using HOMER (version 4.11.1, RRID: SCR_010881) findMotifsGenome.pl (56). Pybedtools (version 0.12.0, RRID: SCR_021018) was used to identify overlapping and unique peaks across treatments for each transcription factor (57). Heatmaps and counts-per-million–normalized bigWig files were generated with deepTools bamCoverage, computeMatrix, and plotHeatmap (46). Peak annotation was performed with ChIPSeeker (version 1.42.1, RRID: SCR_021322; ref. 58). regulaTER (version 0.1.0; bioRxiv 2024.06.29.601318) and RepeatMasker (version open-4.0.5, RRID: SCR_012954) were used to annotate peaks with transposable elements (TE). Gene tracks were created using GViz (version 1.50.0, RRID: SCR_024239; ref. 59). Gene Ontology (GO) analysis for STAT3 and p65 CUT&RUN was performed via chipenrich (version 2.30.0; ref. 60).

### MicroRNA qRT-PCR

Total RNA was isolated from OVCAR3 cell pellets using the RNeasy Mini Kit (Qiagen, 74106) according to the manufacturer’s protocol. cDNA was amplified using primers specific for miR-21-5p and miR-21-3p, using the Taqman MicroRNA Reverse Transcription Kit (Thermo Fisher Scientific, 4366596) as per the manufacturer’s instructions, followed by RT-qPCR using the FastStart Essential DNA Probe Master (Roche, 06402682001). Relative microRNA (miRNA) expression was calculated using the Pfaffl method, with *RNU6B* as the housekeeping gene. Taqman assays used are listed in Supplementary Table S2.

### Antibodies

The following antibodies were used: anti-pSTAT3 [Cell Signaling Technology (CST), 9145, RRID: AB_2491009, Western blot]; anti-STAT3 (CST, 9139, RRID: AB_331757, Western blot and CUT&RUN); anti-p65 (CST, 8242, RRID: AB_10859369, Western blot and CUT&RUN); anti-GAPDH (CST, 5174, RRID: AB_10622025, Western blot); anti–histone H3 (CST, 9717, RRID: AB_331222, Western blot); anti–trimethyl histone H3 (Lys4, CST, 9751, RRID: AB_2616028, Western blot); anti-mouse IgG2a (CST, 61656, RRID: AB_2799613, CUT&RUN); and anti-rabbit IgG (MilliporeSigma, 12-0370, RRID: AB_145841, CUT&RUN).

### Survival data analysis

The survival data analysis of HGSC patient data from GSE18520 was performed using PROGene V2 at https://proggene.ccbb.indianapolis.iu.edu/index.php (61).

### Statistical analysis

Experiments were conducted with at least three biological replicates, except for the CUT&RUN experiment, which used two biological replicates. The results are presented as mean ± SEM unless otherwise noted. Significance was determined using a one-way ANOVA followed by Tukey multiple comparisons test in RStudio (version 2025.09.2+418, RRID: SCR_000432). For all figures, *, *P* ≤ 0.05; **, *P* ≤ 0.01; ***, *P* ≤ 0.001; and ****, *P* ≤ 0.0001. All significant comparisons are shown. Statistical details for each experiment are included in the figures and figure legends.

## Results

### Inhibiting DNA methyltransferases blocks cisplatin-induced OCSC enrichment

**Figure 1.**
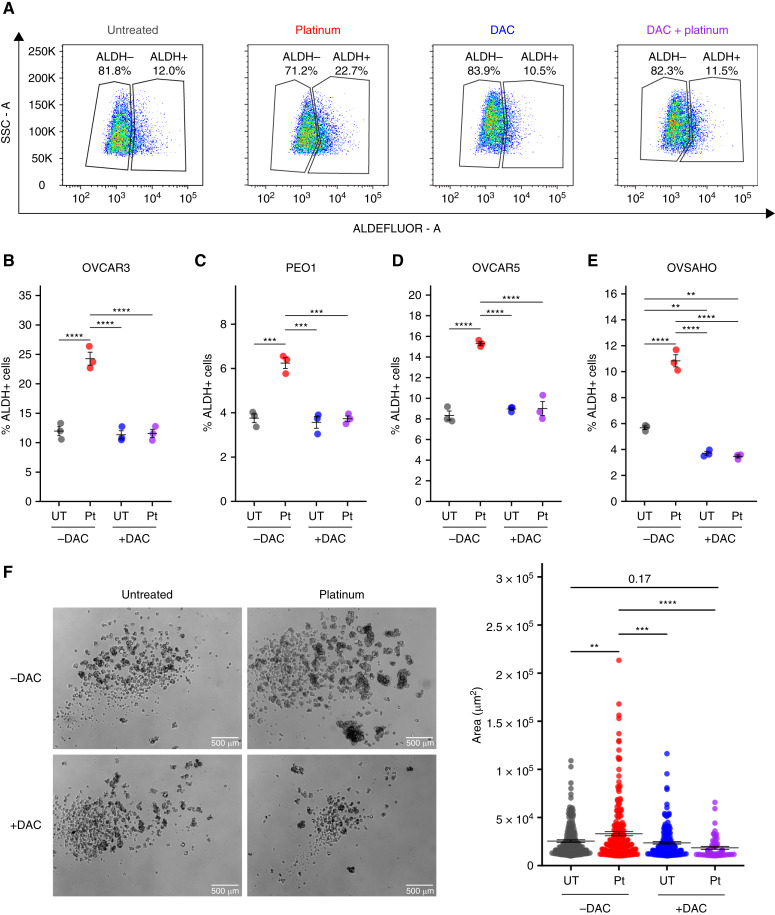
DNMTi treatment prevents platinum-induced enrichment of OCSCs. **A,** ALDEFLUOR assays were performed on OVCAR3 cells treated with 100 nmol/L DAC for 72 hours, followed by 15 μmol/L platinum for the last 16 hours. Gates used to determine the %ALDH+ cells for one biological replicate are shown. **B–E,** Percent ALDH+ (%ALDH+) cells in the indicated cell lines, as determined by flow cytometry. Cells were untreated or treated with their respective IC_50_ doses of platinum (Pt) alone or in combination with 100 nmol/L DAC. Lines indicate mean ± SEM. Each dot represents a biological replicate (*N* = 3). **F,** Spheroid formation assay on OVCAR3 cells pretreated with platinum (7.5 μmol/L, 3 hours) alone or in combination with DAC (100 nmol/L, 72 hours) and then cultured for 7 days. Each point indicates a spheroid. All spheroids from three biological replicates (*N* = 3) are included. The graph shows the mean area ± SEM for all spheroids across three biological replicates. Significance is determined by one-way ANOVA and Tukey honestly significant difference test with **, *P* ≤ 0.01; ***, *P* ≤ 0.001; ****, *P* ≤ 0.0001. Pt, platinum; UT, untreated.

Although we and others have shown that platinum treatment increases the percentage of ALDH+ (%ALDH+) cells in ovarian cancer (9, 13) and DNMTi treatment reduces the %ALDH+ cells and resensitizes platinum-resistant ovarian cancer (5, 16), the underlying mechanisms remain poorly understood. To further evaluate the effect of DNMTi treatment on platinum-induced OCSC enrichment, HGSC cell lines were treated with platinum alone or in combination with DNMTi DAC, and then the ALDH+ OCSC population was analyzed using the ALDEFLUOR assay. DEAB, an ALDH inhibitor, served as an internal control for each treatment group. Consistent with previous findings (9, 13, 24), the %ALDH+ cells increased after platinum alone, whereas %ALDH+ levels remained similar to baseline when cells were treated with DAC alone or with both agents (Fig. 1A–E; Supplementary Fig. S1A).

Next, to functionally evaluate the effect of combining DAC and platinum on OCSC survival and growth, we conducted spheroid formation assays using pretreated OVCAR3 cells grown in stem cell media. Spheroids from platinum-treated OVCAR3 cells were larger than those from untreated cells (Fig. 1F). Similarly, spheroids from OVCAR5 and OVSAHO cells pretreated with platinum showed increased viability compared with untreated cells (Supplementary Fig. S1B and S1C). DAC alone did not affect spheroid formation compared with untreated cells. However, the combination of DAC and platinum reduced spheroid growth and viability relative to platinum-treated cells (Fig. 1F; Supplementary Fig. S1B and S1C). Overall, these results indicate that combining DAC with platinum blocks platinum-induced OCSC enrichment.

### Inflammatory and IFN signaling pathways are enriched in response to platinum and DNMTi

**Figure 2.**
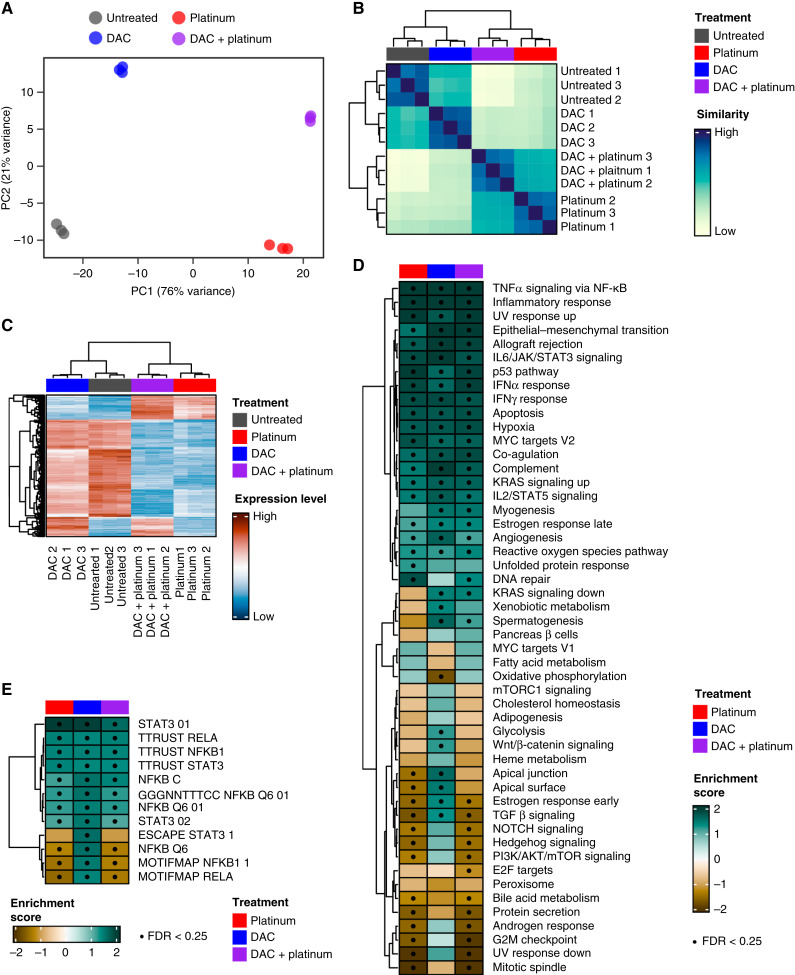
Transcriptomic analysis of OVCAR3 cells treated with platinum alone or in combination with DNMTi treatment. OVCAR3 cells were either untreated or treated with 15 μmol/L platinum for 16 hours, with or without 100 nmol/L DAC for 72 hours. Samples were then collected and subjected to RNA-seq (*N* = 3). **A,** Principal component analysis (PCA) plot of RNA-seq samples. Clustering of samples by (**B**) similarity in expression profiles or (**C**) expression of the 1,000 most variable genes. Heatmaps display the normalized enrichment scores of (**D**) hallmark and (**E**) STAT3 and NF-κB target gene sets for each treatment group relative to the untreated.

To identify pathways altered by platinum and DNMTi treatment in HGSC, we performed RNA-seq on OVCAR3 and PEO1 cells treated with platinum alone or in combination with DAC. Principal component analysis distinguished transcriptomic profiles across treatment groups (Fig. 2A; Supplementary Fig. S2A). Hierarchical clustering based on transcriptomic similarities grouped cells treated with DAC alone with untreated cells. Conversely, cells treated with platinum, either alone or in combination with DAC, clustered together (Fig. 2B; Supplementary Fig. S2B). Similar clustering patterns were observed when using the top 1,000 most variable genes (Fig. 2C; Supplementary Fig. S2C), suggesting that platinum was the primary driver of transcriptional differences. DNMTi treatment is known to upregulate gene expression, and more genes were upregulated than downregulated in DAC-treated compared with untreated cells in both cell lines (Supplementary Fig. S2D and S2E), as expected.

GSEA using MSigDB Hallmark gene sets revealed that IFN and inflammatory responses were the most enriched pathways in OVCAR3 and PEO1 cells treated with platinum and/or DAC compared with untreated cells (Fig. 2D; Supplementary Fig. S2F; Supplementary Table S3). Because platinum and DNMTi have been shown to upregulate type I IFNs and IFN-stimulated genes (ISG; refs. 18, 62), we examined their expression in more detail in OVCAR3 cells. *IFNB1* and most ISGs were upregulated by DAC and remained unchanged after platinum treatment alone (Supplementary Fig. S2G). In contrast, *IFNA1* expression was increased by platinum but not by DAC alone. Interestingly, the antiviral gene *IFIT3* and the cytosolic RNA sensor *DDX58* (RIG1) showed opposite patterns: their expression decreased in response to platinum and increased (*IFIT3*) or remained unchanged (*DDX58*) with DAC alone (Supplementary Fig. S2G).

### Platinum and DNMTi differentially activate NF-κB and STAT3

**Figure 3.**
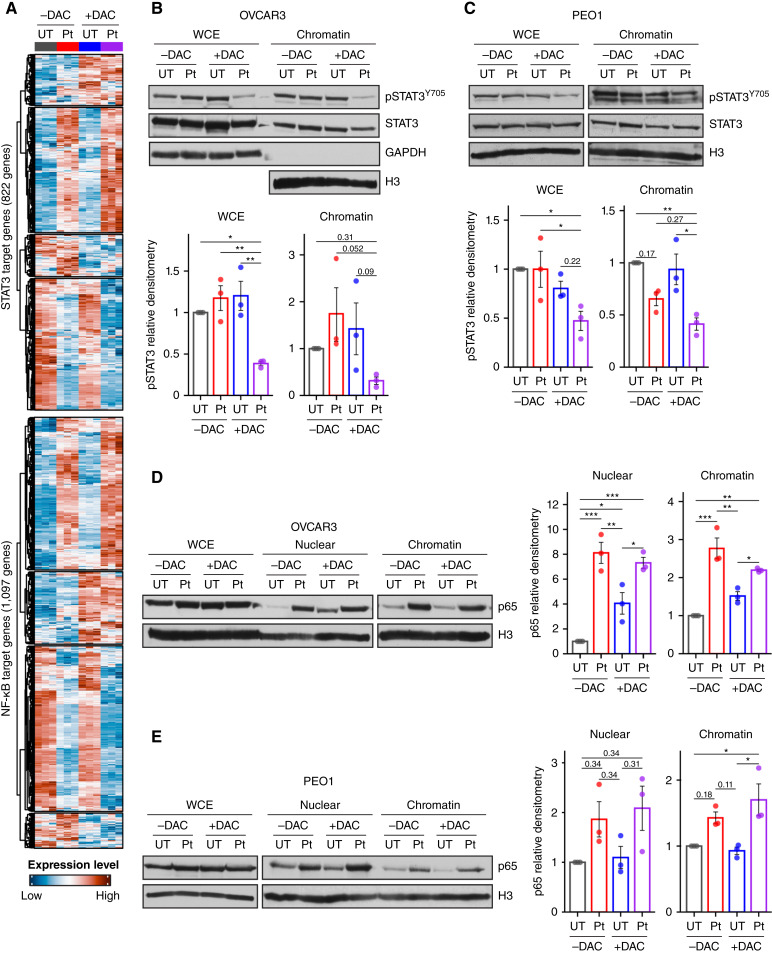
Platinum and DNMTi treatments differentially activate STAT3 and NF-κB in ovarian cancer cells. **A,** Heatmap of expression levels of genes curated from STAT3 and NF-κB target gene sets for each sample in the RNA-seq dataset. **B–E,** Western blot of whole-cell (WCE), nuclear, and chromatin lysates prepared from OVCAR3 and PEO1 cells treated with their respective IC_50_ doses of platinum for 16 hours, with or without 100 nmol/L DAC for 72 hours. The graphs show mean densitometric analysis ± SEM for pSTAT3 (*N* = 3) and p65 (*N* = 3) relative to the loading control (GAPDH for OVCAR3 WCE; total H3 for PEO1 WCE, as well as nuclear and chromatin lysates). Significance is determined by one-way ANOVA and the Tukey honestly significant difference test, with *, *P* ≤ 0.05; **, *P* ≤ 0.01; ***, *P* ≤ 0.001. Pt, platinum; WCE, whole-cell extraction; UT, untreated.

Among the highly enriched inflammatory pathways were the NF-κB and STAT3 signaling pathways (Fig. 2D; Supplementary Fig. S2F; Supplementary Table S3). Because NF-κB and STAT3 are transcription factors linked to tumorigenesis, including stemness in ovarian cancer (24–27), additional GSEA was performed using NF-κB and STAT3 target gene sets. This analysis further demonstrated enrichment of these pathways in the treated groups relative to the untreated group in both OVCAR3 and PEO1 cells (Fig. 2E; Supplementary Fig. S2H; Supplementary Table S4). In addition, expression levels of genes curated from the gene sets in 2E demonstrated that platinum and DAC treatment differentially regulated expression of NF-κB and STAT3 target genes (Fig. 3A), leading us to hypothesize that differences in gene expression resulted from differential activation of NF-κB and STAT3 by these agents in HGSC cells. The level of phosphorylated STAT3 (pSTAT3) at tyrosine 705 was used as a marker for STAT3 activation. There was no change in total pSTAT3 levels in OVCAR3 or PEO1 cells treated with platinum or DAC alone; however, pSTAT3 levels decreased when cells were treated with a combination of platinum and DAC (Fig. 3B and C). Furthermore, pSTAT3 bound to chromatin decreased in the combination treatment compared with untreated or single treatments (Fig. 3B and C), suggesting that DAC combined with platinum reduced STAT3 activation. Next, nuclear localization of the p65 subunit, a marker of NF-κB activation, was examined. Increased levels of nuclear p65 and p65 bound to chromatin were observed after treatment with platinum, either alone or in combination with DAC in OVCAR3 cells (Fig. 3D). However, DAC alone increased p65 nuclear localization but did not induce p65 binding to chromatin (Fig. 3D). A similar trend for platinum and/or DAC inducing p65 nuclear localization and chromatin binding was observed in PEO1 cells (Fig. 3E). Overall, these data demonstrate that whereas single treatment with platinum or DAC has no effect on STAT3 activation in HGSC cells, the combination of platinum and DAC significantly reduces STAT3 activation. Furthermore, platinum treatment induces NF-κB activation and increases chromatin binding, regardless of DAC presence.

### Both NF-κB and STAT3 are essential for platinum-induced OCSC enrichment

**Figure 4.**
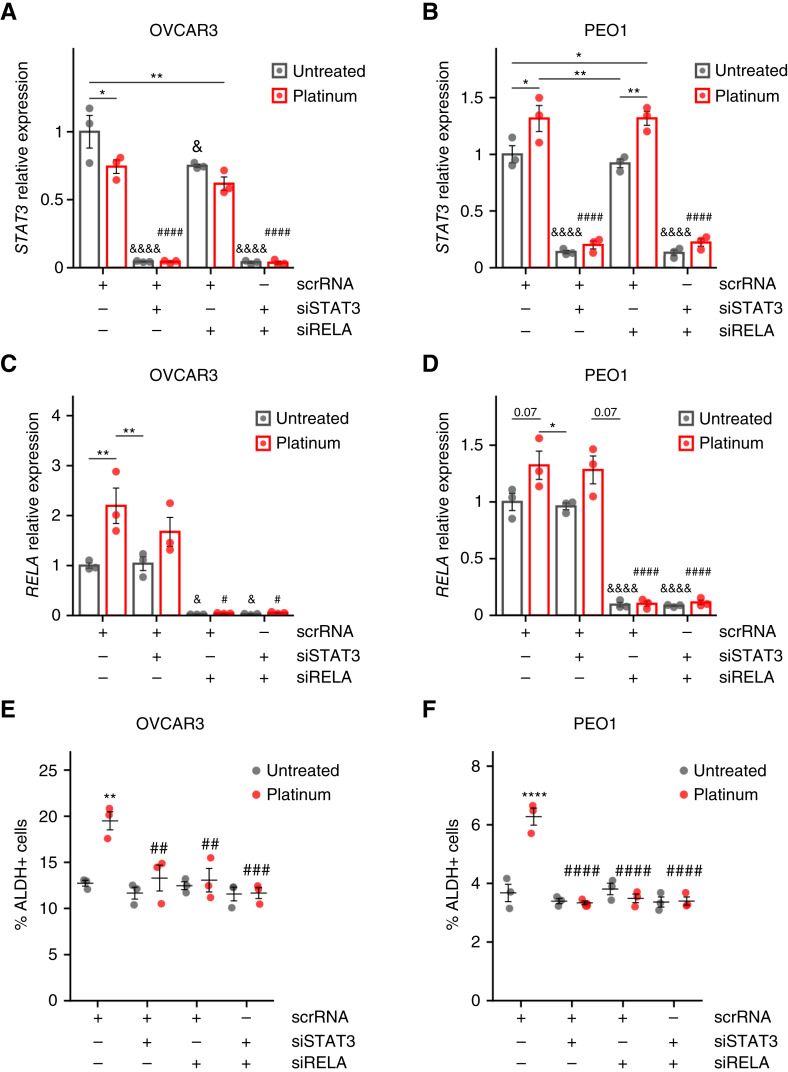
Both STAT3 and NF-κB are required for platinum-induced OCSC enrichment. Relative gene expression of *STAT3* in (**A**) OVCAR3 and (**B**) PEO1 cells treated with scrambled RNA (scrRNA) control and STAT3 and/or RELA (p65) KD (siSTAT3, siRELA, respectively), either untreated or treated with IC_50_ dose of platinum for 16 hours (*N* = 3). Relative gene expression of *RELA* (p65) in (**C**) OVCAR3 and (**D**) PEO1 cells treated as in **A**. Graphs show mean expression ± SEM (*N* = 3). %ALDH+ cells in (**E**) OVCAR3 and (**F**) PEO1 cells treated as in **A** (*N* = 3). Lines indicate mean ± SEM. Each dot represents a biological replicate (*N* = 3). Significance is determined by one-way ANOVA and Tukey honestly significant difference test. For all platinum vs. untreated comparisons, *, *P* ≤ 0.05; **, *P* ≤ 0.01; ****, *P* ≤ 0.0001. For all untreated scrRNA vs. KD cells, &, *P* ≤ 0.05; &&&&, *P* ≤ 0.001. For cisplatin-treated scrRNA vs. KD cells, #, *P *≤ 0.05; ##, *P* ≤ 0.01; ###, *P* ≤ 0.001; ####, *P* ≤ 0.0001.

Based on our findings that STAT3 remained active at the same level as in untreated cells after platinum treatment, as indicated by its phosphorylation levels, and that NF-κB was activated by the treatment (Fig. 3B–E), we tested the hypothesis that the presence of both stemness pathways is necessary for platinum-induced OCSC enrichment. siRNA-mediated KD of STAT3 and/or the NF-κB subunit p65 (*RELA*) had a modest effect on the gene expression of the other in both OVCAR3 and PEO1 cells, as well as on its protein activation in OVCAR3 cells (Fig. 4A–D; Supplementary Fig. S3). However, although p65 and/or STAT3 KD did not alter baseline levels of ALDH+ cells, the platinum-induced increase in %ALDH+ cells was blocked (Fig. 4E and F), demonstrating that both NF-κB and STAT3 are required for platinum-induced OCSC enrichment.

### DMNTi-induced secreted factors are insufficient to block platinum-induced OCSC enrichment

**Figure 5.**
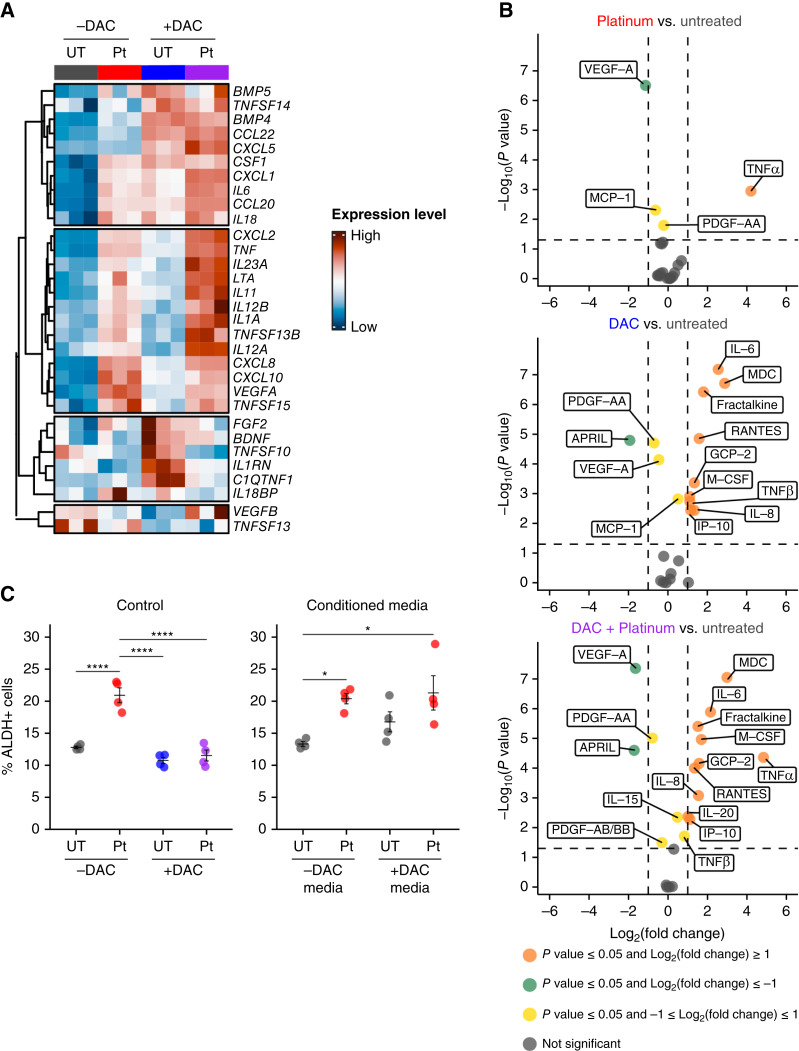
Secretome induced by DNMTi alone is insufficient to prevent platinum-induced OCSC enrichment. **A,** Heatmap of expression levels of cytokine genes curated from STAT3 and NF-κB target gene sets for each sample in the RNA-seq dataset. **B,** Volcano plots of secretome analysis of media collected from OVCAR3 cells treated with 15 μmol/L platinum for 16 hours, with or without 100 nmol/L DAC for 72 hours (*N* = 3). **C,** Naïve OVCAR3 cells were directly treated with platinum (15 μmol/L for 16 hours) and/or DAC (100 nmol/L for 72 hours; control), or incubated in media collected from untreated or DAC-treated cells for 48 hours, with 15 μmol/L platinum added in the last 16 hours (Conditioned media), followed by ALDEFLUOR assay. Lines indicate mean ± SEM. Each dot represents a biological replicate (*N* = 4). Significance was determined by one-way ANOVA and Tukey honestly significant difference test, with *, *P* ≤ 0.05; ****, *P* ≤ 0.0001. Pt, platinum; UT, untreated.

NF-κB and STAT3 regulate the expression of cytokines involved in inflammatory responses (63). Analysis of NF-κB and STAT3 target cytokine genes from the gene sets in 2E revealed distinct expression patterns across treatments, with expression of most cytokines increasing in treated cells (Fig. 5A). Because DAC and platinum caused differential activation of NF-κB and STAT3 (Fig. 3B–E), we hypothesized that DAC and platinum treatments produce distinct cytokine profiles in HGSC cells. To test this hypothesis, media from OVCAR3 cells treated with platinum alone or combined with DAC were collected for secretome analysis. Platinum, with or without DAC, induced secretion of TNFα relative to untreated conditions (Fig. 5B; Supplementary Fig. S4; Supplementary Table S5). Interestingly, DAC treatment induced the secretion of nine additional cytokines, regardless of the presence of platinum. Additionally, secretion of VEGFA and APRIL was decreased by platinum and DAC, respectively (Fig. 5B; Supplementary Fig. S4; Supplementary Table S5).

Cytokines are known to play essential roles in tumorigenesis, including regulating CSCs (64). To investigate the effect of DNMTi-induced cytokine secretion on platinum-induced OCSC enrichment, naïve OVCAR3 cells were grown in conditioned medium collected from untreated or DAC-treated cells and then exposed to platinum. As expected, platinum alone increased %ALDH+ cells, which was prevented by DAC in the control groups without conditioned media (Fig. 5C, control). However, media from DAC-treated cells did not prevent the increase in %ALDH+ cells after platinum treatment compared with untreated media (Fig. 5C, conditioned media). Together, these data demonstrate that DNMTi-induced cytokine secretion alone is insufficient to prevent platinum-induced enrichment of OCSCs and further indicate that an intracellular mechanism may be involved.

### Platinum and DNMTi treatment alter binding of STAT3 and NF-κB to the genome

**Figure 6.**
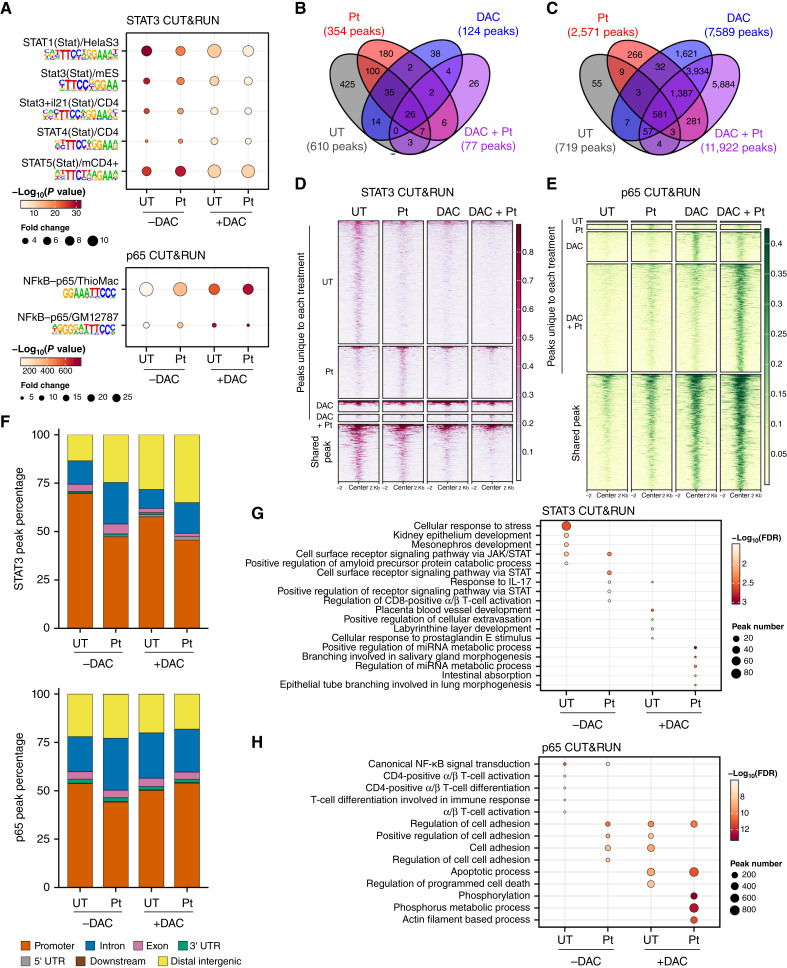
Platinum and DNMTi alter STAT3 and p65 binding. **A,** Top enriched motifs from HOMER analysis of STAT3 (top) and p65 (below) CUT&RUN data from OVCAR3 cells treated with 15 μmol/L platinum for 16 hours, with or without 100 nmol/L DAC for 72 hours (*N* = 2). Venn diagrams show (**B**) STAT3 and (**C**) p65 CUT&RUN overlaps across treatments. Metagenomic heatmaps show (**D**) STAT3 and (**E**) p65 unique and shared peaks. **F,** Bar graphs show the percentage of peaks annotated to each genomic feature for STAT3 and p65. Dot plots show GO enrichment analysis of (**G**) STAT3 and (**H**) p65 CUT&RUN peaks using ChIP-Enrich. Pt, platinum; UT, untreated; UTR, untranslated region.

Based on the observed differential effect of platinum and DNMTi on STAT3 and NF-κB activation and chromatin binding (Fig. 3B–E), it was of interest to identify genomic binding sites for STAT3 and NF-κB across different treatments. We performed CUT&RUN assays for STAT3 and NF-κB subunit p65 on OVCAR3 cells treated with platinum, with or without DAC. HOMER motif analysis showed that STAT3 and p65 peaks were strongly enriched for STAT and NF-κB/p65 motifs, respectively (Fig. 6A), confirming the specificity of the antibodies for each transcription factor. After DAC treatment (alone or combined with platinum), the number of STAT3 peaks decreased (Fig. 6B), whereas p65 peaks increased relative to untreated and platinum alone (Fig. 6C). STAT3 and p65 bound different genomic regions in response to each treatment (Fig. 6D and E), with some regions shared across conditions. In these shared regions, DAC reduced STAT3 binding and increased p65 binding, especially when combined with platinum. Examination of the genomic locations of the peaks showed that the majority of STAT3 and p65 peaks were in gene promoters, regardless of treatment (Fig. 6F; Supplementary Tables S6 and S7). GO analysis of STAT3 peaks, performed using the ChIP-Enrich method (60), showed enrichment for genes involved in a range of distinct biological processes for each treatment (Fig. 6G; Supplementary Table S8). On the other hand, GO analysis of p65 peaks across treatments revealed enrichment for genes related to immune cell activation and cell adhesion, with greater enrichment in the DAC-treated groups (Fig. 6H; Supplementary Table S8). Overall, these findings suggest that DNMTi and platinum differentially alter STAT3 and NF-κB genomic binding sites and target gene associations.

### Alterations in STAT3 and NF-κB cistromes in response to platinum and/or DNMTi correlate with changes in expression of nearby genes

**Figure 7.**
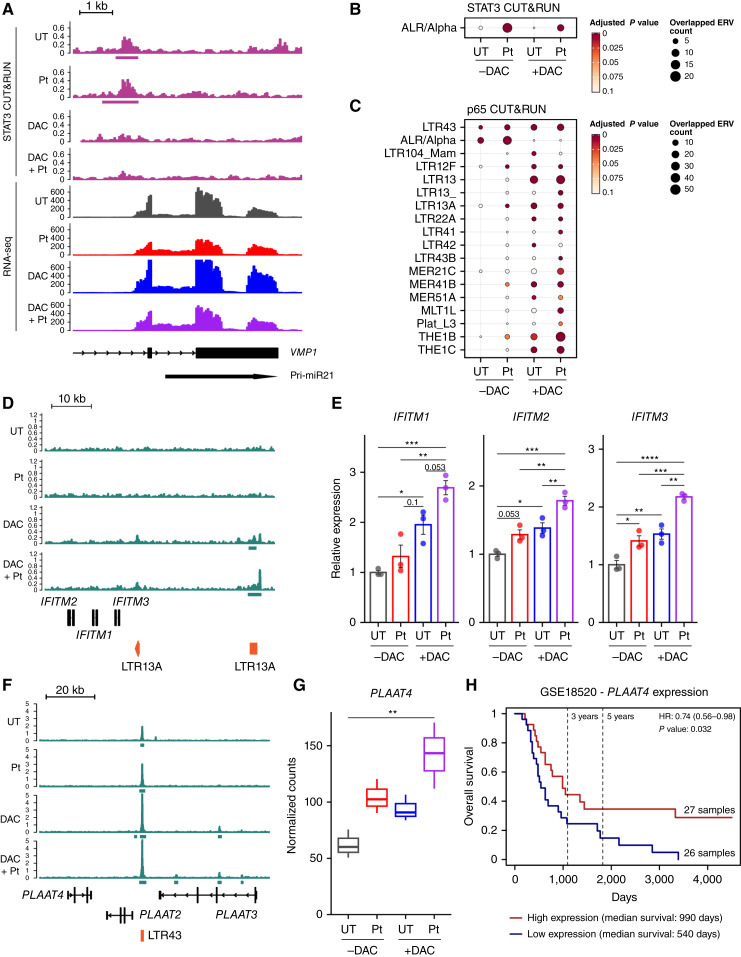
Platinum and DAC differentially alter chromatin binding of STAT3 and p65 to TEs. **A,** Gene tracks showing STAT3 CUT&RUN and RNA-seq read enrichment for the primary transcript of microRNA 21 in OVCAR3 cells. Enrichment of TE members in (**B**) STAT3 and (**C**) p65 peaks as determined by regulaTER. **D,** Gene track showing p65 CUT&RUN peaks at LTR13A and nearby genes. **E,** Relative gene expression of *IFITM1*, *IFITM2*, and *IFITM3* in OVCAR3 cells treated with platinum alone (15 μmol/L for 16 hours) or in combination with DAC (100 nmol/L for 72 hours) was measured by qRT-PCR. The graph shows the mean expression ± SEM (*N* = 3). **F,** Gene track showing p65 CUT&RUN peaks at LTR43 and nearby genes. **G,** Box plot showing normalized read counts of *PLAAT4* in response to various treatments from OVCAR3 RNA-seq data. **H,** Kaplan–Meier survival curve for patients with HGSC from GSE18520, stratified based on *PLAAT4* expression. Significance is determined by one-way ANOVA and Tukey honestly significant difference test: *, *P* ≤ 0.05; **, *P* ≤ 0.01; ***, *P* ≤ 0.001; ****, *P* ≤ 0.0001. Pt, platinum; UT, untreated.

CUT&RUN analysis further revealed that STAT3 binding to intronic and intergenic regions increased after platinum and/or DAC treatment compared with untreated cells (Fig. 6F; Supplementary Table S7). In contrast, p65 binding to introns increased in response to platinum and/or DAC treatment, whereas binding to intergenic regions remained relatively unchanged (Fig. 6F; Supplementary Table S7). Because miRNAs are located in noncoding regions (65) and abnormal miRNA expression has been shown to play a role in CSC (66), we examined the STAT3 and p65 peaks in intronic and intergenic regions to identify nearby miRNAs. Several STAT3-binding peaks were annotated to neighboring miRNAs (Supplementary Table S7), and among them was a peak located in the intron of the *VMP1* gene (vacuole membrane protein-1), upstream of the miRNA-21 (miR-21) primary transcript, in both untreated and platinum-treated groups, but not in DAC-treated samples (Fig. 7A). Additionally, RNA-seq data analysis revealed more aligned reads mapping to the miR-21 primary transcript downstream of the STAT3-binding site after treatment (Fig. 7A), but no reads were mapped to other annotated miRNAs. As STAT3 is known to regulate miR-21 expression (67, 68), and miR-21 has been shown to play a role in ovarian cancer tumorigenesis and platinum resistance (69–71), we examined the expression of the mature miR-21 transcripts, miR-21-5p and miR-21-3p, in OVCAR3 cells treated with platinum with or without DAC to further evaluate the increased aligned reads seen in the RNA-seq data. Expression of miR-21-5p decreased when platinum was combined with DAC, whereas there was a trend toward increased miR-21-3p expression with platinum alone but not when combined with DAC (Supplementary Fig. S5A).

In addition to microRNAs, TEs are frequently located in noncoding regions such as introns and intergenic regions (72). Because some TEs contain transcription factor–binding motifs (72), we explored whether STAT3 and p65 bind to TEs in response to different treatments. We observed an enrichment of STAT3 peaks in ALR/Alpha satellite repeats following platinum treatment, regardless of the presence of DAC (Fig. 7B; Supplementary Fig. S5B; Supplementary Table S9). Interestingly, p65 peaks were enriched in ALR/Alpha satellite repeats in untreated and platinum-treated groups but not in DAC-treated groups (Fig. 7C; Supplementary Fig. S5C; Supplementary Table S10). Conversely, p65 peaks showed enrichment in the long terminal repeats (LTR, also known as ERVs) following DAC alone or combined with platinum. LTRs have been shown to contain transcription factor–binding sites, thereby acting as alternative promoters and enhancers that regulate the expression of nearby genes (73). Some genes near LTR-bound p65 peaks in DAC-treated samples were associated with infection responses, including the *IFITM* family, whose expression increased in OVCAR3 cells treated with both platinum and DAC relative to untreated and single-treatment groups (Fig. 7D and E). Other genes associated with LTR-bound p65 included inflammatory cytokines TNFα (*TNF*) and TNFβ (*LTA*), which showed increased secretion in response to treatments (Supplementary Fig. S5D; Fig. 4C). An increase in p65 binding to LTR43 was also observed near *the PLAAT4* gene following DAC treatment (Fig. 7F). *PLAAT4* has been shown to promote terminal differentiation in keratinocytes (74), as well as act as a tumor-suppressor (75–77). RNA-seq analysis revealed upregulation of *PLAAT4* in OVCAR3 and PEO1 cells treated with both platinum and DAC compared with untreated and single-treatment groups (Fig. 7G; Supplementary Fig. S5E). Interestingly, analysis of HGSC patient data from GSE18520 indicated that higher *PLAAT4* expression was associated with improved survival (Fig. 7H). Similar trends were also observed for *IFITM1* and *IFITM2* genes (Supplementary Fig. S5F and S5G). Overall, these results suggest that combining platinum with a DNMTi increases p65/NF-κB binding to LTRs and potentially alters the expression of nearby genes.

## Discussion

OCSC driving relapse and platinum resistance presents a challenge to improving patient ovarian cancer outcomes. In this study, we show that combining platinum with DNMTi treatment blocks platinum-induced enrichment of OCSCs. Based on preclinical data demonstrating that DNMTi treatment resensitizes platinum-resistant ovarian cancer cells (5, 16), previous clinical trials focused on treating patients with platinum-resistant ovarian cancer with DNMTis to resensitize their cancers to platinum (21, 22). Promising trends in phase II trials with improved 6-month progression-free survival (PFS) were observed, despite not meeting the primary statistical endpoint for overall median PFS compared with standard chemotherapy (22). Blocking enrichment of OCSCs at an earlier stage provides rationale for using DNMTis with platinum in the neoadjuvant setting. We hypothesize that DNMTi treatment will be more effective in this setting because it prevents the development of resistance by blocking OCSC enrichment rather than reversing resistance once it has already developed.

Our data demonstrate that either DAC treatment alone or KD of STAT3 and/or NF-κB prevents the increase in ALDH+ cells following platinum treatment, whereas baseline levels remain unchanged. Notably, these observations align with our previous work, which showed that OXPHOS inhibition also blocked the platinum-induced increase in ALDH+ cells without affecting baseline levels (13). These findings suggest that DAC disrupts mechanisms that drive the increase in ALDH+ cells after platinum and that these processes are distinct from those that maintain baseline ALDH+ cells. DAC is a cytosine analog that incorporates into newly synthesized DNA strands, thereby trapping the DNMT protein and leading to its degradation (78). Based on this mechanism of action, cells must be dividing for DAC to work, and it takes multiple days for DAC treatment to inhibit DNMTs. Therefore, DAC treatment needs to occur prior to platinum treatment in our experiments to ensure DNMT inhibition. Consequently, we cannot use DAC to test whether DNMT inhibition during platinum treatment only is sufficient to block the platinum-induced increase in %ALDH+ cells.

Our analysis of ovarian cancer cell gene expression reveals that both platinum and DNMTi upregulate IFN response and other inflammatory pathways, consistent with previous findings (18, 62). Based on our new observation that IFN and ISG expression vary across treatments, we suggest that DNMTi treatment and platinum exert different effects on these pathways. In addition to their roles in fighting infections, IFNs and IFN responses can be either pro- or anti-stemness in cancer, depending on the cancer type (79–81). However, the role of IFNs in regulating stemness in ovarian cancer remains largely unknown, and further investigations are needed to determine whether the distinct IFN responses induced by platinum and DNMTis contribute to the different effects of these treatments on OCSCs.

In this study, we show that DNMTi treatment and platinum not only differentially activate NF-κB and STAT3 transcription factors but also affect the binding of these transcription factors to chromatin, likely altering target gene expression. The roles of NF-κB and STAT3 in regulating stemness and differentiation basally and in response to treatment in ovarian cancer and other cancers have been well established (24–27). Although NF-κB and STAT3 are known to be essential for platinum-induced enrichment of ALDH+ OCSCs (24, 26), it was previously unknown whether the transcription factors need to be present simultaneously for this enrichment to occur. Our findings demonstrate for the first time that the presence of both NF-κB and STAT3 is required for platinum to increase ALDH+ OCSCs.

Additionally, we demonstrated that DNMTi treatment induces greater cytokine secretion than platinum alone but that this secretion alone is insufficient to block platinum-induced OCSC enrichment. Given that activation of STAT3 has been shown to reduce NF-κB binding to antitumor immune response genes (82), and only NF-κB activity is high when DNMTi treatment is combined with platinum, it is likely that the combination shifts cancer cells toward a proinflammatory phenotype, perhaps mediated by cytokines. However, based on our results, these secreted cytokines likely are not the mechanism by which DNMTi treatment blocks the platinum-induced increase in OCSCs. A limitation of our media exchange experiment is that some secreted factors, such as Wnt factors, may not have been stable enough in the conditioned media to exert their effects on the naïve cells. Interestingly, many of the detected cytokines are known immune-cell attractants: IL-8, RANTES, GCP-2, IP-10, fractalkine, and MDC (64). As ovarian cancer is known to have an immunosuppressive environment (83, 84), it is possible that combining DNMTi with platinum *in vivo* will induce the cancer cells to secrete immune cell–recruiting cytokines, resulting in activation of an antitumor response that will also improve responses to platinum therapy. Further investigations are required to explore the effects of DNMTis and platinum on immune cell recruitment.

Finally, when DNMTi treatment is combined with platinum, it seems to regulate gene expression at least in part by reducing STAT3 binding at miRNA promoters while allowing NF-κB binding to demethylated LTR regions. Because STAT3 is known to regulate miR-21 expression (67, 68), and CSCs across different cancer types have high miR-21 levels (85–87), it is plausible that combining DNMTi with platinum reduces STAT3 activation and chromatin binding, thereby lowering miR-21 levels and preventing OCSC enrichment. Our CUT&RUN data reveal increased NF-κB binding to LTRs in response to DNMTi. Given that DNMTi-induced demethylation of LTRs has been previously observed in ovarian cancer (88), it is possible that NF-κB accesses these regions due to DNMTi-induced DNA hypomethylation. Although DNMTi treatment alone has minimal effects on the expression of genes near these NF-κB–bound LTR sites, their expression is significantly upregulated with the addition of platinum. Interestingly, genes like *PLAAT4* and *IFITM* have been shown to promote differentiation (74, 76, 89–92), and their higher expression correlates with improved clinical outcomes in patients with HGSC. Together, these results suggest that a possible mechanism by which combining DNMTi with platinum prevents OCSC enrichment is by inducing NF-κB binding to LTRs, thereby upregulating the expression of neighboring genes involved in differentiation. However, the exact molecular mechanism of these gene expression changes remains unknown and requires further investigation.

Overall, we demonstrate that combining DNMTi with platinum-based therapy prevents platinum-induced enrichment of OCSCs in HGSC. We propose a possible mechanism by which DNMTi blocks platinum-induced OCSC enrichment by altering the activation and binding of NF-κB and STAT3 to target genes. Collectively, our findings provide preclinical evidence supporting the use of DNMTi in combination with platinum-based therapy to prevent OCSC enrichment, thereby reducing relapse and resistance and improving patient outcomes.

## Supplementary Material

Supplementary Figure S2 Transcriptomic analysis of OVCAR3 and PEO1 cells treated with platinum alone or in combination with DNMTi.

Supplementary Figure S3 Knocking down STAT3 or p65 did not affect the activity of the other.

Supplementary Figure S4 DNMTi induces greater cytokine secretion in OVCAR3 than platinum alone.

Supplementary Figure S5 Platinum and DAC differentially alter chromatin binding of STAT3 and p65 to transposable elements.

Supplementary Table S1 Curated NF-KB and STAT3 target gene sets.

Supplementary Table S2 qRT-PCR primers.

Supplementary Table S3 GSEA analysis using MSigDB Hallmark gene sets.

Supplementary Table S4 GSEA analysis using curated NH-KB and STAT3 target gene sets.

Supplementary Table S5 Secretome analysis of media from OVCAR3 treated with platinum and/or DAC.

Supplementary Tabls S6 Called peaks from IDR analysis of STAT3 and p65 CUT&RUN data.

Supplementary Table S7 Peak annotation of STAT3 and p65 CUT&RUN peaks.

Supplementary Table S8 ORA analysis with ChIPenrich on STAT3 and p65 CUT&RUN data.

Supplementary Table S9 STAT3 CUT&RUN ERV annotation.

Supplementary Table S10 p65 CUT&RUN ERV annotation.

Supplementary Figure S1 DNMTi prevents platinum-induced enrichment of OCSCs.

## Data Availability

The bulk RNA-seq and CUT&RUN datasets are available via the NCBI Gene Expression Omnibus (RRID: SCR_005012) under accession numbers GSE319609, GSE319610, and GSE332978. The data and code used to generate the graphs in this article are available on GitHub (https://github.com/truc-vuong/OC_STAT3_and_NF-kB_paper). All other raw data generated in this study are available upon request to the corresponding author.
