# Supplementary material for: DNA Methyltransferase Inhibition Prevents Platinum-Induced Ovarian Cancer Stem Cell Enrichment
Source: Cancer Res Commun. 2026 Jul 20;6(7):1721–37. doi: 10.1158/2767-9764.CRC-26-0149 (PMC13381740; doi:10.1158/2767-9764.CRC-26-0149)
Supplement: Supplementary Figure S2 — Transcriptomic analysis of OVCAR3 and PEO1 cells treated with platinum alone or in combination with DNMTi. [file crc-26-0149_supplementary_figure_s2_suppsf2.pdf]

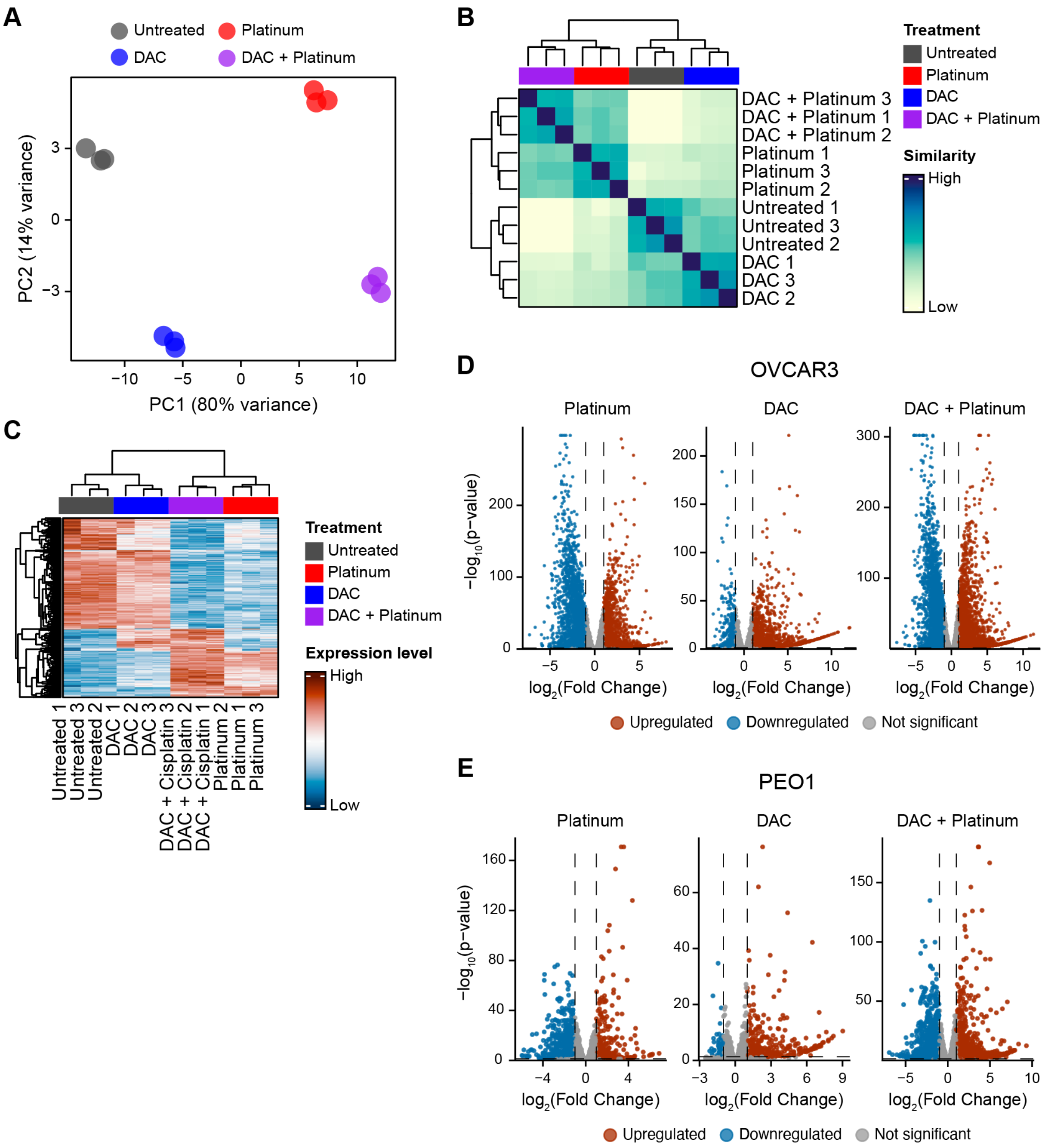

A
Untreated
Platinum
DAC
DAC + Platinum
PC2 (14% variance)
PC1 (80% variance)
B
Treatment
Similarity
High
Low
DAC + Platinum 3
DAC + Platinum 1
DAC + Platinum 2
Platinum 1
Platinum 3
Platinum 2
Untreated 1
Untreated 3
Untreated 2
DAC 1
DAC 3
DAC 2
C
Expression level
Untreated 1
Untreated 3
Untreated 2
DAC 1
DAC 2
DAC 3
DAC + Cisplatin 3
DAC + Cisplatin 2
DAC + Cisplatin 1
Platinum 2
Platinum 1
Platinum 3
D
OVCAR3
E
PEO1
Platinum
DAC
DAC + Platinum
−log10(p−value)
log2(Fold Change)
Upregulated
Downregulated
Not significant

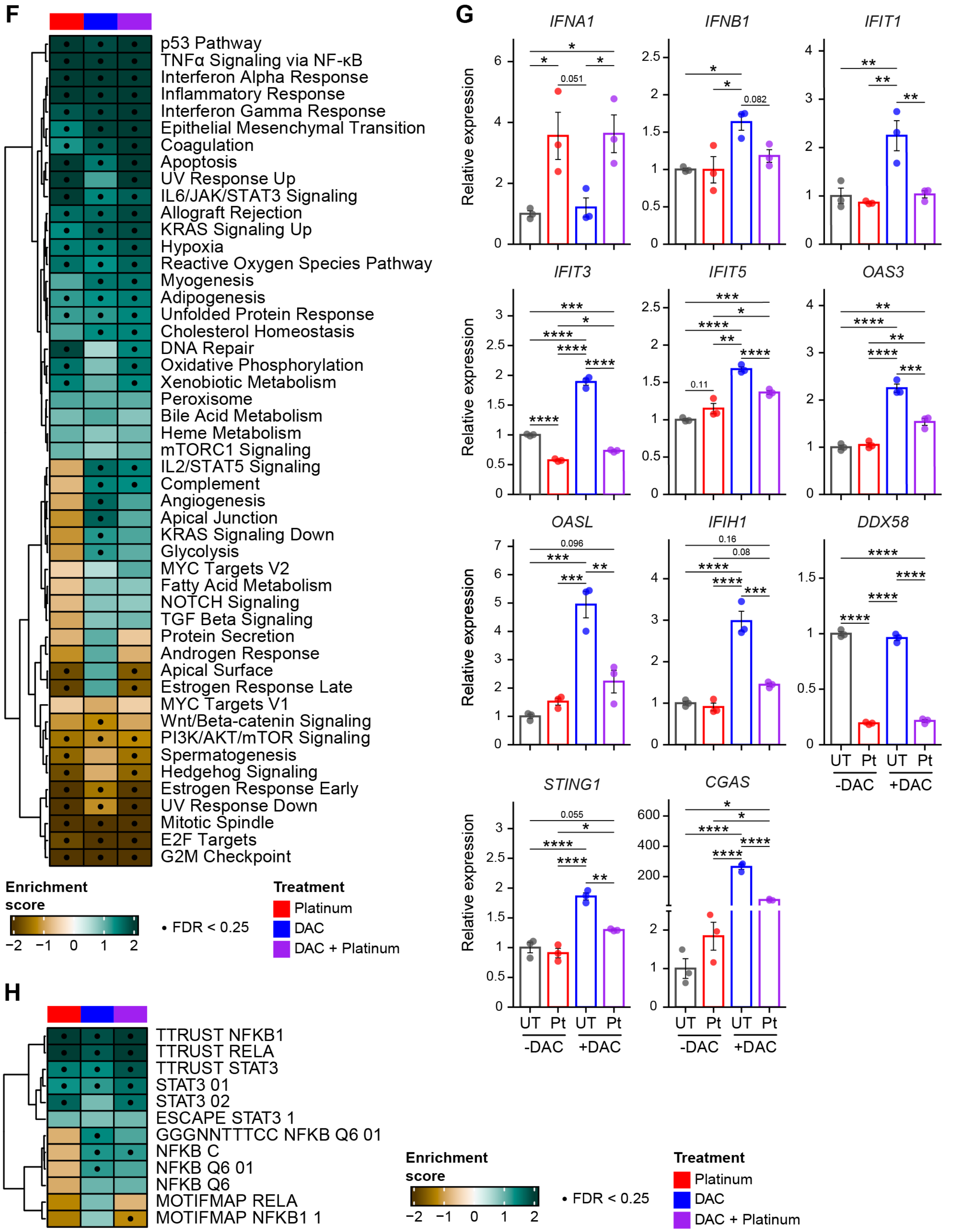

Supplementary Figure S2. Transcriptomic analysis of OVCAR3 and PEO1 cells treated with platinum alone or in combination with DNMTi. (**A**) Principal component analysis (PCA)

plot of RNA-seq samples of PEO1 cells either untreated or treated with 13 μM platinum for 16 hours, with or without 100 nM DAC for 72 hours (N = 3). Clustering of samples by (**B**) similarity in expression profiles or (**C**) expression of the 1000 most variable genes. Volcano plot of differentially expressed genes in (**D**) OVCAR3 and (**E**) PEO1 cells in response to platinum and/or DAC relative to untreated. Dashed lines indicate $p \leq 0.05$ and $| \log_2(\text{fold change}) | > 1$. Heatmaps display the normalized enrichment scores of (**F**) hallmark, and (**H**) STAT3 and NF-κB target gene sets for each treatment group relative to the untreated. (**G**) Relative gene expression by qRT-PCR of type I IFNs: *IFNA1* and *IFNB1*; ISGs: antiviral genes *IFIT1*, *IFIT3, IFIT5*, *OAS3*, and *OASL*; genes involved in the cytosolic RNA-sensing pathway *IFIH1* (MDA5) and *DDX58* (RIG1); and genes involved in the cytosolic DNA-sensing pathway *STING1* and *CGAS* in OVCAR3 cells treated with 15 μM platinum for 16 hours, with or without 100 nM DAC for 72 hours. Graphs show mean expression ± SEM (N = 3). Significance is determined by one-way ANOVA and Tukey HSD test: * $p \leq 0.05$, ** $p \leq 0.01$, *** $p \leq 0.001$, **** $p \leq 0.0001$.
