# Supplementary material for: DNA Methyltransferase Inhibition Prevents Platinum-Induced Ovarian Cancer Stem Cell Enrichment
Source: Cancer Res Commun. 2026 Jul 20;6(7):1721–37. doi: 10.1158/2767-9764.CRC-26-0149 (PMC13381740; doi:10.1158/2767-9764.CRC-26-0149)
Supplement: Supplementary Figure S3 — Knocking down STAT3 or p65 did not affect the activity of the other. [file crc-26-0149_supplementary_figure_s3_suppsf3.pdf]

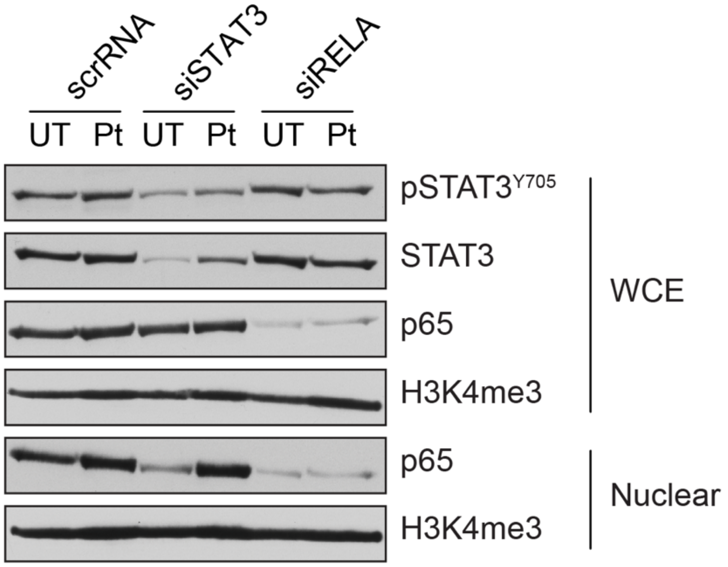

Supplementary Figure S3. Knocking down STAT3 or p65 did not affect the activity of the other. Western blot analysis of whole-cell lysate (WCE) and nuclear extracts of OVCAR3 cells transfected with scramble RNA (scrRNA) control, siSTAT3, or siRELA, followed by treatment with 15 μM platinum for 16 hours.
