# Supplementary material for: DNA Methyltransferase Inhibition Prevents Platinum-Induced Ovarian Cancer Stem Cell Enrichment
Source: Cancer Res Commun. 2026 Jul 20;6(7):1721–37. doi: 10.1158/2767-9764.CRC-26-0149 (PMC13381740; doi:10.1158/2767-9764.CRC-26-0149)
Supplement: Supplementary Figure S4 — DNMTi induces greater cytokine secretion in OVCAR3 than platinum alone. [file crc-26-0149_supplementary_figure_s4_suppsf4.pdf]

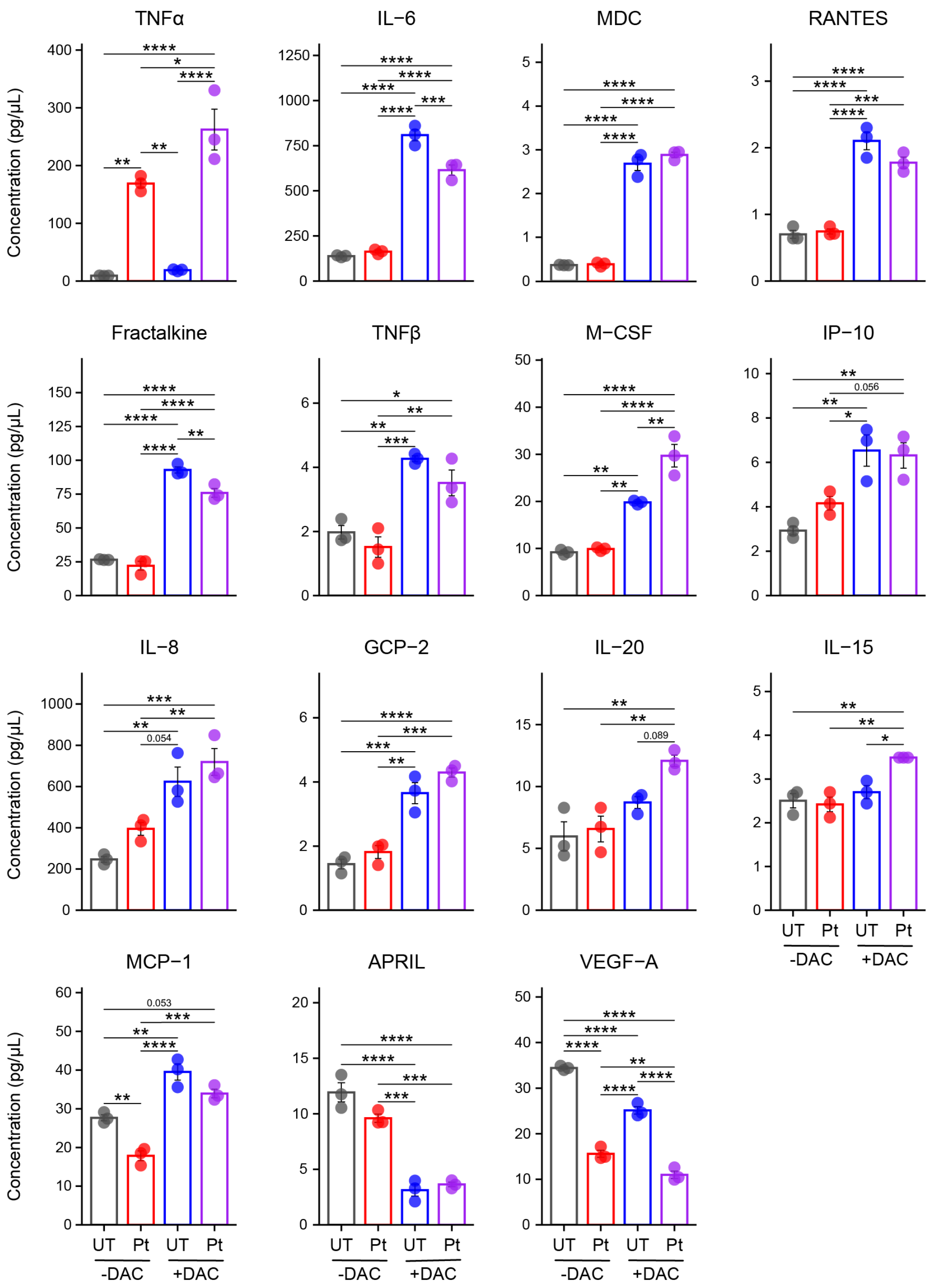

Supplementary Figure S4. DNMTi induces greater cytokine secretion in OVCAR3 than platinum alone. Concentrations of differentially secreted cytokines after treating OVCAR3 cells with 15 μM platinum for 16 hours, with or without 100 nM DAC for 72 hours. Graphs show mean concentration ± SEM (N = 3). Significance is determined by one-way ANOVA and the Tukey HSD test, with * $p \leq 0.05$, ** $p \leq 0.01$, *** $p \leq 0.001$, and **** $p \leq 0.0001$.
