# Supplementary material for: DNA Methyltransferase Inhibition Prevents Platinum-Induced Ovarian Cancer Stem Cell Enrichment
Source: Cancer Res Commun. 2026 Jul 20;6(7):1721–37. doi: 10.1158/2767-9764.CRC-26-0149 (PMC13381740; doi:10.1158/2767-9764.CRC-26-0149)
Supplement: Supplementary Figure S5 — Platinum and DAC differentially alter chromatin binding of STAT3 and p65 to transposable elements. [file crc-26-0149_supplementary_figure_s5_suppsf5.pdf]

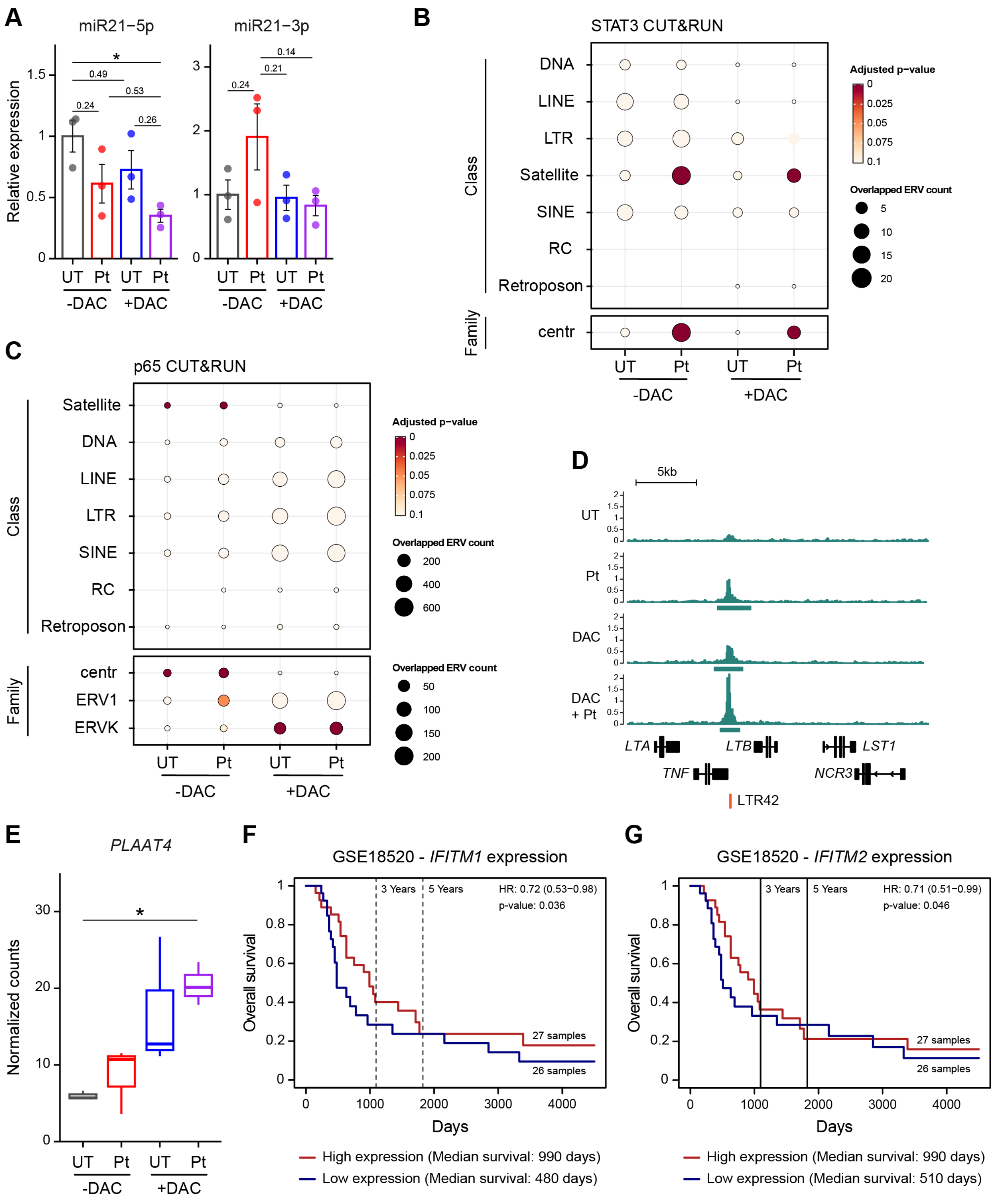

Supplementary Figure S5. Platinum and DAC differentially alter chromatin binding of STAT3 and p65 to transposable elements. (**A**) Relative gene expression of mature mir21-5p and mir21-3p in OVCAR3 cells treated with 15 μM platinum for 16 hours, with or without

100 nM DAC for 72 hours. Graphs show the mean expression ± SEM (N = 3). Annotations of (**B**) STAT3 and (**C**) p65 CUT&RUN data to transposable element classes and families using regulaTER. (**D**) Gene track showing p65 CUT&RUN at LTR42 and nearby genes. (**E**) Box plot showing normalized read counts of *PLAAT4* in response to various treatments from PEO1 RNA-seq data. Kaplan-Meier survival curve for HGSC patients from GSE18520, stratified based on (**F**) *IFITM1* and (**G**) *IFITM2* expression. Significance is determined by one-way ANOVA and the Tukey HSD test, with * $p \leq 0.05$.
