# Supplementary material for: DNA Methyltransferase Inhibition Prevents Platinum-Induced Ovarian Cancer Stem Cell Enrichment
Source: Cancer Res Commun. 2026 Jul 20;6(7):1721–37. doi: 10.1158/2767-9764.CRC-26-0149 (PMC13381740; doi:10.1158/2767-9764.CRC-26-0149)
Supplement: Supplementary Figure S1 — DNMTi prevents platinum-induced enrichment of OCSCs. [file crc-26-0149_supplementary_figure_s1_suppsf1.pdf]

A

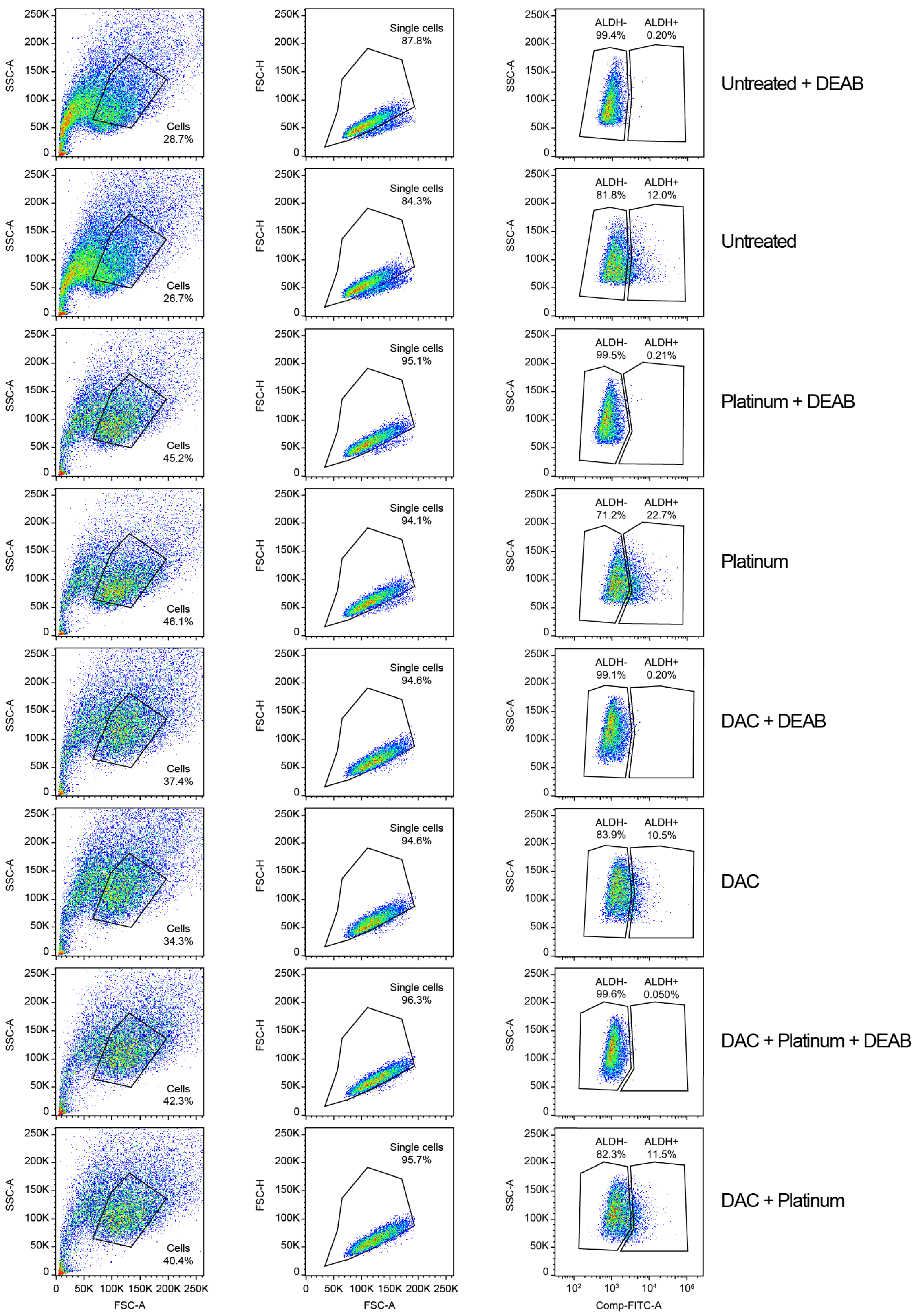

Cells 28.7%
Single cells 87.8%
ALDH- 99.4%
ALDH+ 0.20%
Untreated + DEAB
Cells 26.7%
Single cells 84.3%
ALDH- 81.8%
ALDH+ 12.0%
Untreated
Cells 45.2%
Single cells 95.1%
ALDH- 99.5%
ALDH+ 0.21%
Platinum + DEAB
Cells 46.1%
Single cells 94.1%
ALDH- 71.2%
ALDH+ 22.7%
Platinum
Cells 37.4%
Single cells 94.6%
ALDH- 99.1%
ALDH+ 0.20%
DAC + DEAB
Cells 34.3%
Single cells 94.6%
ALDH- 83.9%
ALDH+ 10.5%
DAC
Cells 42.3%
Single cells 96.3%
ALDH- 99.6%
ALDH+ 0.050%
DAC + Platinum + DEAB
Cells 40.4%
Single cells 95.7%
ALDH- 82.3%
ALDH+ 11.5%
DAC + Platinum
SSC-A
FSC-A
FSC-H
Comp-FITC-A

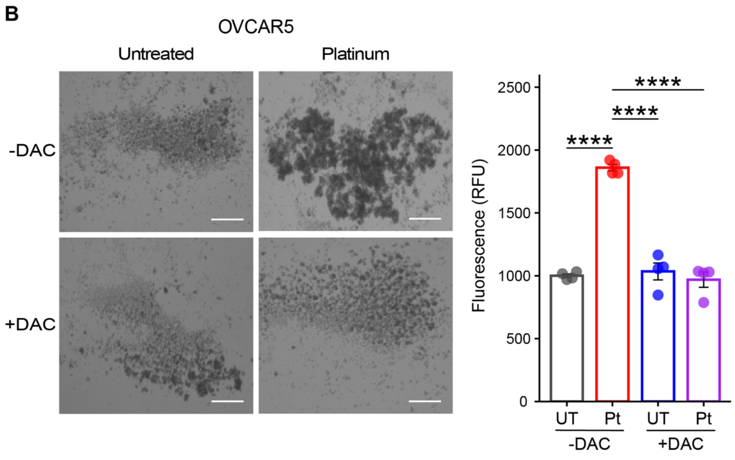

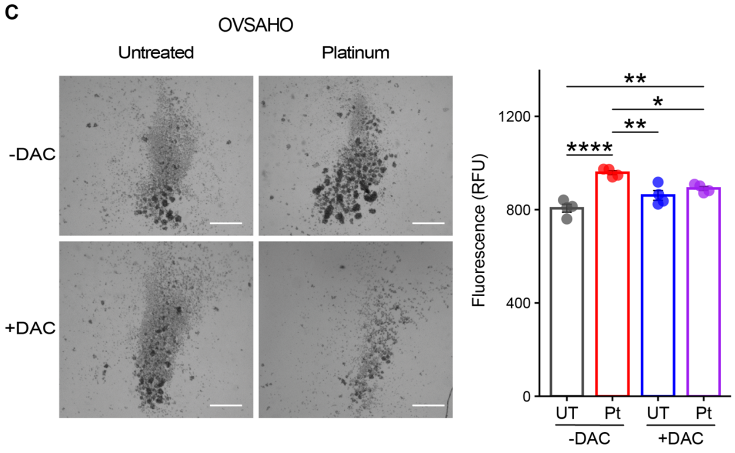

Supplementary Figure S1. DNMTi prevents platinum-induced enrichment of OCSCs. (**A**) The plots show the gating strategy used to determine the percentages of ALDH- and ALDH+ cells in OVCAR3 cells treated with 15 μM platinum for 16 hours, with or without 100 nM DAC for 72 hours, in the presence of the ALDH inhibitor DEAB or the ALDEFLUOR reagent. Spheroid formation assay on (**B**) OVCAR5 and (**C**) OVSAHO cells pre-treated with half of their respective IC50 doses of platinum (Pt) for 3 hours alone or in combination with 100 nM DAC for 48 hours, then cultured for 14 days. The graphs show mean relative fluorescence units (RFU) ± SEM across four biological replicates (N = 4). Significance is determined by one-way ANOVA and Tukey HSD test, with ** $p \leq 0.01$, *** $p \leq 0.001$, **** $p \leq 0.0001$.
